# Annotations

**Published:** 1891-02-21

**Authors:** 


### Annotations.

**What is Madness?**

A definition of madness is badly wanted; but what is wanted still more is the erasure of certain old notions from the medical mind and from the mind of the general public. It has been decided after a careful inquiry by medical experts and a jury that Mr. Francis Henry Paget, of Birstall, Leicestershire, a country gentleman, is unable to manage his own affairs, and the management of them has been placed in the hands of Mrs. Ada Mary Walker, of Maunby Hall, Yorkshire, his daughter and heiress. In order to bring about this result the only course open to the friends of Mr. Paget was to charge him with lunacy, and to take the usual steps to prove the charge. Mr. Paget, on the other hand, had friends, both legal and medical, who undertook to defend his sanity. It is discreditable, and discreditable in the highest degree, both to the law and to the medicine of Great Britain that such a charge was the only way of dealing with a case like that of Mr. Paget; and it is equally discreditable to medicine that so much cross-swearing as has taken place should have been possible. A hundred terms might be suggested which would define such a case better than lunacy or madness; and which would not only be therefore more true to fact and science, but also less offensive to an afflicted person and to his family and friends. Mr. Paget, like thousands of others, is in a state of mental incapacity. It is evident that he has neither suicidal nor homicidal mania. He is quite harmless, except in so far as that he has a faculty for wasting money. Why then should we not find terms and definitions for such cases, which, whilst scientifically classifying them, shall justify exactly so much loss of freedom of action as they warrant? The want of originality, mental power, and professional courage among those medical specialists who deal with mental diseases is positively amazing. We talk of our advanced science and our new knowledge! The truth is that for practical purposes we are in many particulars hardly on a level with the Egyptian civilisation of three thousand years ago.

**Dr. Rentoul is sarcastic.**

Dr. Robert Rentoul, of Liverpool, is a well-intentioned man of considerable physical dimensions and energic lung power. He is also endowed with a *cacoëthes scribendi*, and makes large demands on the patience of editors and the space of their papers. Some months ago Dr. Rentoul was good enough to send us several communications bearing upon a scheme he had for the establishment of a public medical service. There were certain good points in the scheme, and we, therefore, published some of Dr. Rentoul's letters, and made sundry comments thereupon, doing what lay in our power to forward his views. The scheme, we believe, was a failure, owing not so much to its positive demerits, as to its absence of simplicity and its lack of easy workableness. A little later in the year Dr. Rentoul took up another scheme, and was again so kind as to address us on the subject, with a view to our help. We presume that he had done the same to other medical papers. But Dr. Rentoul's new departure, which consisted in running a-muck against the Midwives' Registration Bill and all who supported it, did not commend itself to our judgment. On the contrary, we felt sure that the doctor was taking a mistaken view of things, and that his torpedo-like activity was calculated to do harm to a large number of poor lying-in women, and also to injuriously affect the reputation and the best interests of the medical profession. We, therefore, took steps to oppose these peculiar vagaries; of course, with courtesy, but also with decision. Whereupon the adversary, apparently unaccustomed to good-humoured fighting accompanied by blows straight from the shoulder, incontinently gets angry, and rounds upon his former friend; and, like Mrs. Gamp after her seventeenth glass of gin and water, "rises up and denounces" us in the *Liverpool Daily Post*. According to this satirical deserter, we are now "An issue known as The Hospital," and "although posing as a semi-medical paper," we do "not in any respect represent the profession." Fie, fie, doctor! This is not good manners; and it certainly is not good fighting form. We do not "pose" as a "semi-medical paper" at all. There is nothing "semi" about us—at least, in intention. We always strive to be *totus, teres atque rotundus*. Moreover, we do not profess to represent the medical profession, nor do we know of any other medical paper that makes any such preposterous assumption. Medical papers represent their editors and their staff, and as far as possible the science and art of medicine. They would be insane if they professed to represent the members of the medical profession. May we make a suggestion to Dr. Rentoul? It is that he shall learn to fight with good temper: and that when he receives a whacking blow from an opponent he shall not go into an inconsequent fit of hysterics, but return the compliment with round, sound, and knock-down logic. Above all, let him avoid sarcasm! It is a game which cannot be played successfully without a keen wit.

**Threatened Closing of a Hospital Ward.**

The Treasurer of the North-West London Hospital, Mr. George Herring, has addressed a communication to the Committee, under date January 21st, 1891, which ought to be taken into earnest consideration, not only by the Committee of the hospital, but also by all persons who make it part of their duty to support medical charities. Mr. Herring states that the hospital has incurred debts amounting to more than £2,000, and that it has only £700 to meet them. He says that under these circumstances expenditure must be immediately curtailed; and he proposes the closing of the men's ward. Mr. Herring further states that owing to the poverty of the neighbourhood the hospital now treats nearly a thousand out-patients every week. This number will also have to be reduced in consequence of straitened means. One more fact ought to be stated before any comments and criticisms are entered upon: it is this—that though the hospital is situated in London, where food and all other necessaries are expensive, the cost of each in-patient per week for food, drugs, and all other things necessary for his care and cure is only twenty-five and sixpence. We are asked by the Committee of the hospital to make these facts publicly known; and also to offer any comments thereupon which seem to be just and called for by the occasion. It so happens that the present writer is quite familiar with the district where the North-West London Hospital is situated. He himself sees every week thirty or forty out-patients in that very district, a considerable portion of whom are men. That the people who live around the hospital are poor he knows well. The greater portion of his own out-patients owe their illnesses more to want of food than to any other cause. So much is this the case that he sometimes says to his medical friends that the principal remedies he prescribes for his out-patients are "Iron and Soup Tickets." It is manifest, therefore, that the people who live in that particular part of North-west London called Kentish Town are not able either to pay their own doctors or to subscribe to the hospital. It is proposed to close the men's ward. That we think would be nothing short of a calamity to the neighbourhood. There is no other hospital within about two miles, the nearest being the Great Northern Central in the Holloway Road on the north side, St. Mary's at Paddington on the west, and University College in Gower Street on the south. For hospital purposes, a distance of two miles in London is equivalent to ten or twelve miles in the country. We say, with all the measured calmness of a judicial or a scientific statement, that instead of the one men's ward of this hospital being closed, there ought to be at least a dozen additional men's wards opened in the district. We make no attempt to be pathetic in this case. Two facts we are assured of—first, that the work of the North-West London Hospital is very injuriously crippled for want of immediate funds; and, second, that many persons who make philanthropy a duty will hurry to the rescue if they can only be fully informed of the state of the case. We have now done our part. We ask other newspapers to do what they can. But, above all, we urge upon the Committee, the medical staff, and all the officials, the immediate necessity of making new efforts in the face of new circumstances, and the imperative duty of keeping open the men's ward at whatever cost.

